# Simultaneous Recovery of Precious and Heavy Metal Ions from Waste Electrical and Electronic Equipment (WEEE) Using Polymer Films Containing Cyphos IL 101

**DOI:** 10.3390/polym13091454

**Published:** 2021-04-30

**Authors:** Katarzyna Witt, Włodzimierz Urbaniak, Małgorzata A. Kaczorowska, Daria Bożejewicz

**Affiliations:** 1 Faculty of Chemical Technology and Engineering, UTP University of Science and Technology, 3 Seminaryjna Street, 85326 Bydgoszcz, Poland; malgorzata.kaczorowska@utp.edu.pl (M.A.K.); daria.bozejewicz@utp.edu.pl (D.B.); 2 Faculty of Chemistry, Adam Mickiewicz University in Poznan, 8 Uniwersytetu Poznańskiego Street, 61712 Poznan, Poland; Wlodzimierz.Urbaniak@amu.edu.pl

**Keywords:** electronic scrap, metal recovery, polymer films, Cyphos IL 101

## Abstract

In this article, the application of a polymer film containing the ionic liquid Cyphos IL 101 for the simultaneous recovery of precious and heavy metal ions ((Ni(II), Zn(II), Co(II), Cu(II), Sn(II), Pb(II), Ag(I), Pd(II), and Au(III)) from waste electrical and electronic equipment (WEEE) is described. The experiments were performed for solutions containing metal ions released from computer e-waste due to leaching carried out with concentrated nitric(V) acid and aqua regia. It was found that the applied polymer film allows for the efficient recovery of precious metals (98.9% of gold, 79.3% of silver, and 63.6% of palladium). The recovery of non-ferrous metals (Co, Ni, Cu, Zn, Sn, and Pb) was less efficient (25–40%). Moreover, the results of the performed sorption/desorption processes show that the polymer film with Cyphos IL 101 can be successfully used after regeneration to recover metals ions several times.

## 1. Introduction

The dynamic growth in the production of electrical and electronic equipment (EEE) around the world and the numerous innovations in such equipment—e.g., in terms of miniaturization and affordability—have reduced its life, consequently leading to the generation of large amounts of so-called e-waste (WEEE) [1,2,3,4]. The amount of WEEE generated varies between countries and depends mainly on economic and technological developments, consumption levels, and the availability of EEE equipment, and it is expected to increase systematically regardless of the discrepancies [5]. Electronic scrap is diverse; it includes spent products used in the production of integrated circuits, PCB, connectors, wiring, etc., as well as batteries and fluorescent lamps [6,7]. WEEE contains many different hazardous components that can be released during improper storage or processing, posing a threat to human health and the environment [8], as well as valuable precious metals, such as gold or silver, the recovery of which is desirable [7,8,9,10]. For example, conductive elements of printed circuit boards include, among others, gold, silver, tin, and zinc [11,12]. Therefore, the development of methods for reducing of the amount of electronic scrap—enabling its reuse, recycling, and various forms of recovery—is extremely important not only for the protection of the natural environment (energy saving, reduction of water and air pollution), but also for economic reasons [13,14,15,16]. The limited amounts of valuable metals in ores and the economic, political, and social problems associated with their mining also contribute to the search for new solutions/methods for the recovery of various metals from e-waste, which can be treated as their secondary source [12,17].

Methods for recovering valuable metals from e-waste, as used today, are based primarily on physical processes—in which the metal fraction is separated from non-metals using specific differences in their properties (e.g., magnetism, electrical conductivity, density, etc.)—and on chemical processes, which are utilized in pyrometallurgical, hydrometallurgical, or biotechnological techniques [18,19]. Hydrometallurgical methods are characterized by their high accuracy, predictability, simplicity of control, and possibility of planning the metal recovery processes at various scales. Therefore, they are of great interest and will probably play a key role in the management of waste electronic and electrical equipment in the future [18,20]. However, such recovery processes are usually complex, and they involve many unit operations, ranging from disassembly, grinding, and physical separation of components to leaching, which leads to the transfer of the desired substances into aqueous solutions, followed by extraction [11].

An alternative to hydrometallurgical processes is the recovery of metals through more environmentally friendly methods, such as modified polymer materials. They have been increasingly used in recent years as an alternative to classic solvent extraction in the recovery of various metal ions from e-waste [21,22]. Because the efficiency of metal recovery processes based on the application of polymer materials depends on the type and properties of the carrier (ion exchanger or compound with complexing properties), intensive research related to the possibility of using various molecules for this purpose [17,23], including commercially available chemicals, such as Cyphos IL 101, Kelex 100, Aliquat 336, and Calix[4]pyrolles [24,25,26,27], has been conducted. Campos et al. [28] tested Cyphos IL 101 for gold recovery from HCl solutions in liquid/liquid extraction systems and immobilized in a biopolymer composite matrix. They reported that gold can be easily desorbed from a loaded ionic-liquid-impregnated resin using thiourea (in HCl solutions), and the resin can be reused for at least four cycles. Vincent et al. [29] immobilized Cyphos IL 101 in capsules prepared by ionotropic gelation in calcium chloride solutions. They used resins containing various amounts of ionic liquid and found that the maximum sorption capacity was different for wet and dry resins (about 177 and 142 mg Pt g^−1^, respectively) and depended on various factors. For example, zinc ions significantly decreased Pt sorption, which was probably due to the competition effect of chloro-anionic Zn species. Platinum can be desorbed from the loaded resin using either nitric acid (5 M) or thiourea (0.1 M in 0.1 M HCl acid solution). Regel-Rosocka et al. [30] investigated an application of phosphonium ionic liquids for removal of Pd(II) ions from aqueous chloride solutions with liquid–liquid extraction and transport across polymer inclusion membranes (PIMs). The authors obtained the highest values of the normalized initial flux for a CTA membrane containing Cyphos IL 101 and a receiving phase: mixture of 0.1 thiourea + 0.5 M HCl. Often, in the PIM separation processes described in the literature, the different metal ions were recovered in experiments based on the utilization of model solutions, of which the composition and properties can easily be changed/controlled. For example, Pospiech [31] applied PIMs containing Cyphos IL 101 and Cyphos IL 104 for the separation of Co(II) and Cu(II) ions from model chlorine ion aqueous solutions (NaCl/HCl) and reported that the selectivity coefficients for cadmium and copper ions decreased with increasing HCl concentration in the source phase. Kozłowski et al. used PIMs equipped with Calix[4]pyrrole derivative as a carrier for the separation of Ag(I) from different nitrate aqueous solutions. They found that the effective transport of silver ions depends on many factors—not only on the concentrations of the carriers and metal ions, but also on the pH and temperature of the source aqueous phase [32]. Because separation processes based on the application of PIMs and other polymer sorbents are influenced by many parameters [24,27], it is extremely important to compare the effectiveness of polymer materials with specific carriers in model and real solutions. Although such studies are carried out, they usually concern the separation of one or two metal ions [33], and many types of e-waste contain several or many different metals. Moreover, even in the case of commercial carriers with known properties, it is uncertain how effective the polymer materials containing such carriers as complexing agents will be in an environment of real composite samples.

In this paper, we present the results of the application of polymer films containing Cyphos IL 101 as a carrier for use in the simultaneous recovery of Pb(II), Ni(II), Zn(II), Co(II), Cu(II), Sn(II), Ag(I), and Au(III) ions from average computer scrap waste.

## 2. Materials and Methods

### 2.1. E-Waste

**Figure 1:**
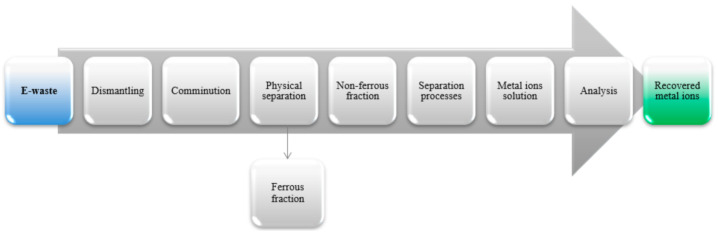
Stages of handling waste electrical and electronic equipment (WEEE).

Treatment of waste electrical and electronic equipment is a complex and challenging process (Figure 1) that involves many unit operations. First, e-waste must be mechanically separated to carry out a leaching process, whereby valuable metal ions can be recovered from aqueous solutions through techniques such as solvent or membrane extraction.

The e-waste was harvested from old computers. Motherboards, compact disc drives (CDDs), and floppy disk drives (FDDs) were removed. In light of metal recovery studies, processors are the most valuable parts of computers because they contain a large amount of pure material that does not require much cleaning.

**Figure 2:**
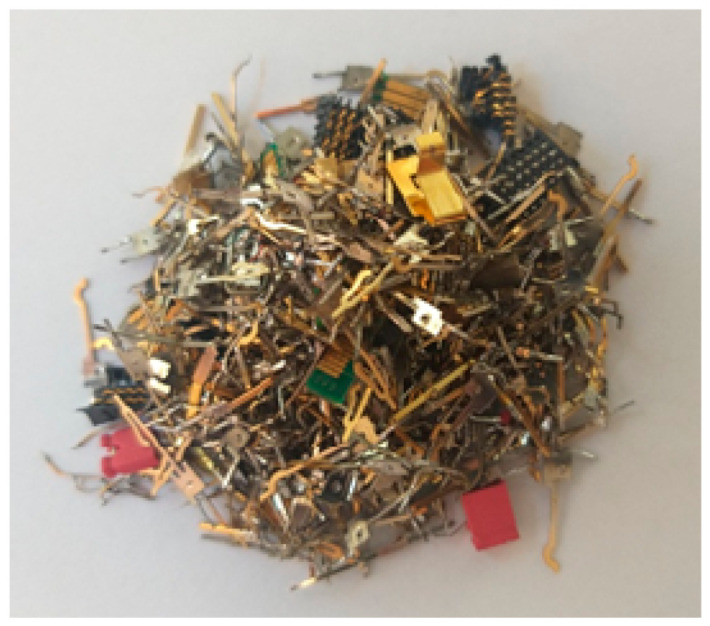
Computer pins used in this research.

The processor pins were separated from the PCBs using a soldering torch. Graphics cards, random-access memory (RAM), and modems containing gold-plated contacts on the PCBs were separated mechanically by cutting them out. The contacts of the video graphics array (VGA) and digital visual interface (DVI) connectors were similarly separated. The computer material harvested in this way was the subject of this study (Figure 2).

### 2.2. Reagents

The suspension of poly(vinyl chloride) (PVC), of an average molecular weight of 72,000, was obtained from ANWIL (Wloclawek, Poland). Tetrahydrofuran (analytical grade) was purchased from Avantor (Gliwice, Poland) and was used without further purification. The phosphonium ionic liquid Cyphos IL 101 and bis(2-ethylhexyl)adipate (DAO) were purchased from Sigma-Aldrich (Poznan, Poland). Concentrated chloric and nitric acids were purchased from Avantor (Gliwice, Poland). The 5 M solution of nitric acid was obtained through dilution of the concentrated acid with distilled water. That solution was standardized against anhydrous sodium carbonate. The “aqua regia” solution was prepared by mixing concentrated nitric acid with concentrated chloric acid in a volume ratio of 1:3.

### 2.3. Preparation of Polymer Films Containing Cyphos IL 101

**Figure 3:**
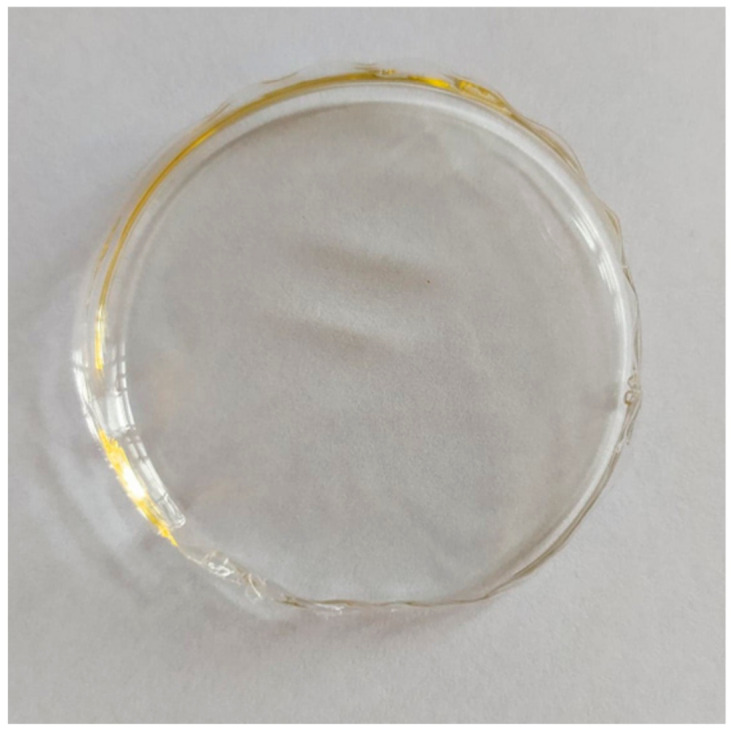
Prepared polymer film.

The polymer films (Figure 3) were prepared by pouring the received solution on a glass. The solution contained 60 wt.% poly(vinylchloride) (PVC) as a support; 20 wt.% bis(2-ethylhexyl)adipate (ADO) as a plasticizer and 20 wt.% a Cyphos IL 101 as an ion carrier were prepared in 10 cm^3^ tetrahydrofuran. After slow evaporation of the solvent for 12 h, the resulting polymer film was peeled off from the glass plate and cut on a few rings with a diameter 4.4 cm. Over the next 12 h, the films were immersed in distilled water. The films were homogeneous, transparent, and flexible, and they had a good strength. The mean thickness of the films was determined and was approximately 0.25 nm.

### 2.4. Preparation of Polymer Films Containing Cyphos IL 101

**Figure 4:**
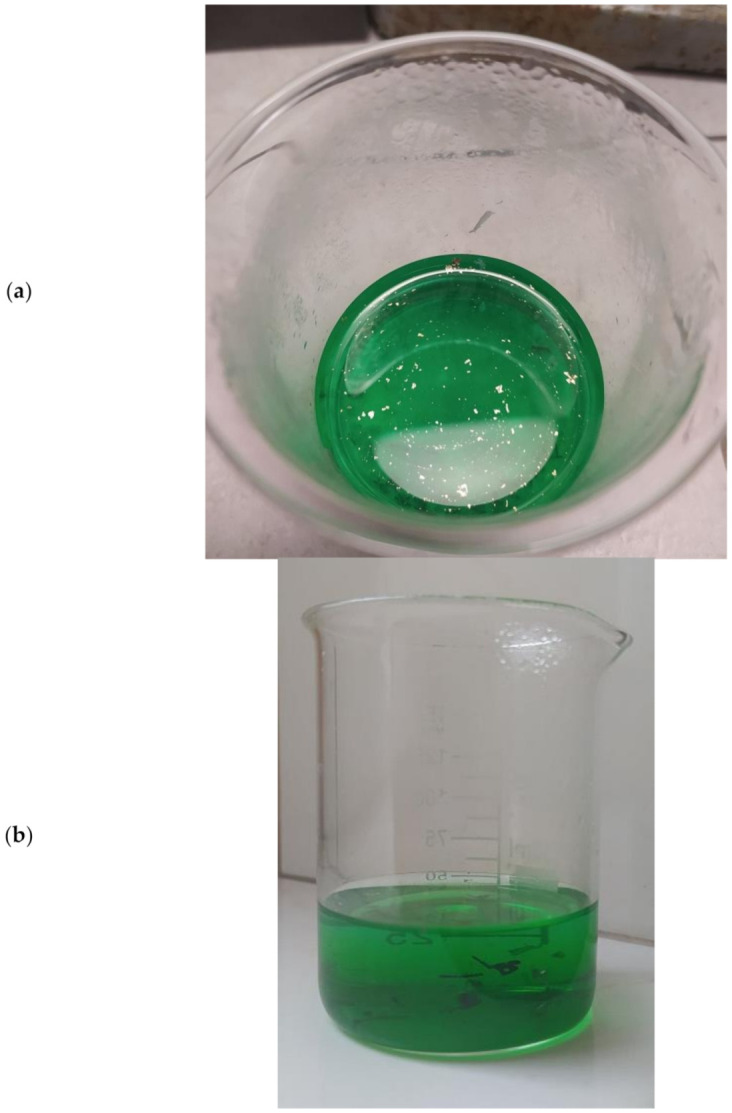

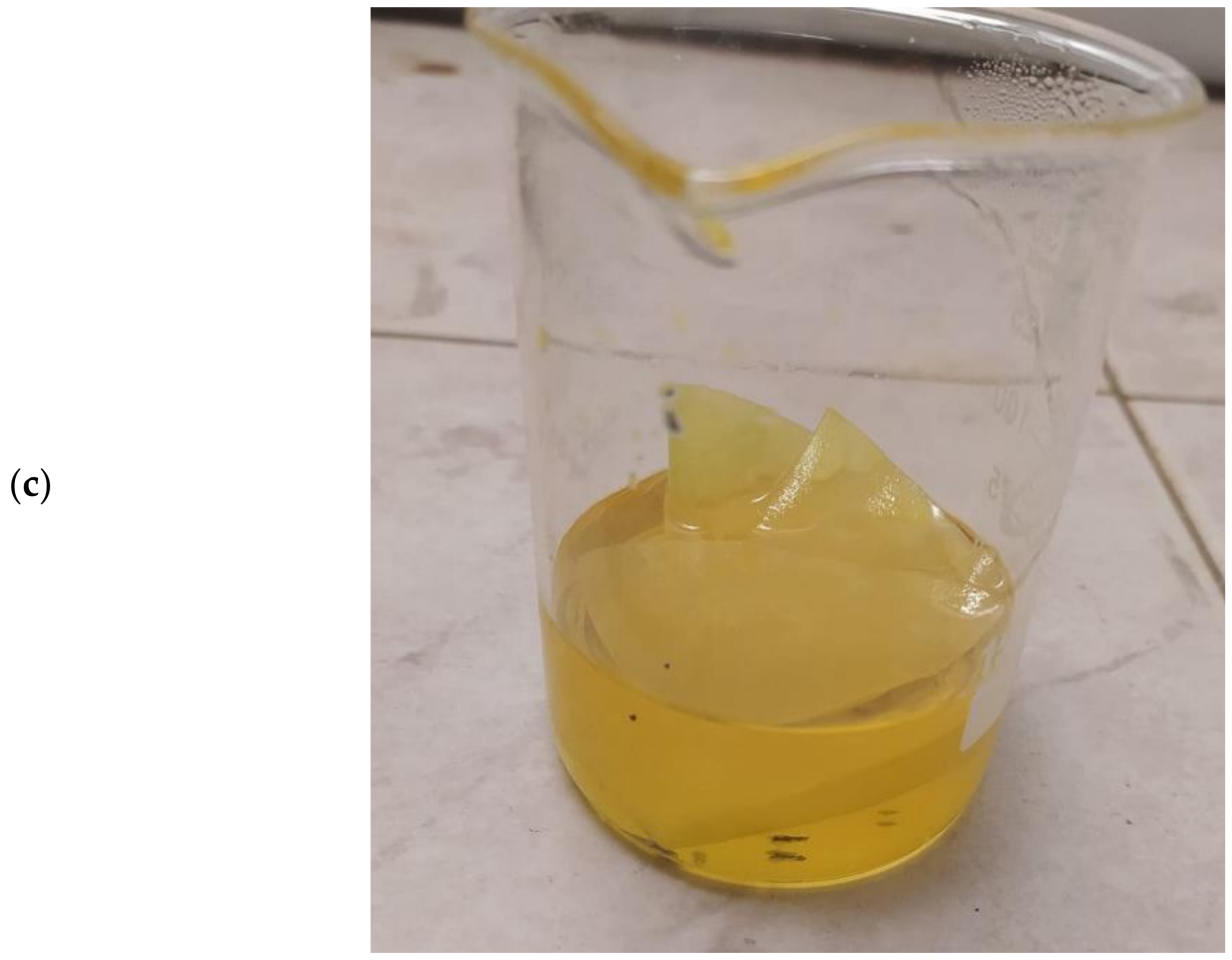
Preparation of computer pin samples for analysis: (**a**) computer pins dissolved in nitric (V) acid—gold flakes visible on the surface; (**b**) computer pins dissolved in nitric (V) acid—visible undissolved pin residues; (**c**)—dissolving of insoluble components in nitric (V) acid residue of computer pins in aqua regia.

Testing of computer pins, which were harvested from e-waste after the pre-treatment described in the “E-waste” subsection (Section 2), involved the following steps (Figure 4a–c):

1. The computer pins were qualitatively analyzed for the presence of metals. This study was performed using a Panalytical XRF Minipal X-ray fluorescence spectrometer.
2. Computer pin samples (0.5 g) were flooded with 50 mL of concentrated (65%) nitric(V) acid and allowed to stand for 24 h. After some time, gold was released on the surface of the solution (Figure 4a). At the bottom, however, a certain amount of undissolved precipitate remained. The gold flakes were separated from the solution through the decantation of the solution from over the undissolved precipitate, and then this solution was centrifugated. In this way, 0.36 mg of gold was obtained.
3. The undissolved precipitate (Figure 4b) mentioned in point 2, together with the small amount of remaining solution, was separated by filtration.
4. To the filter containing precipitate mentioned in point 3, 50 mL of aqua regia was added (Figure 4c). After some time, the filter with the precipitate dissolved completely.

As a result of these operations, two solutions containing metals released from the pins were obtained: a solution of metals in nitric(V) acid (*A*) and a solution of metals in aqua regia (*B*).

### 2.5. Recovery of Metal Ions on Polymer Films Containing Cyphos IL 101

The polymer films obtained as described in the “Preparation of polymer films containing Cyphos IL 101” subsection were used to recover metals from two solutions prepared in line with the “Leaching e-waste” subsection.

**Figure 5:**
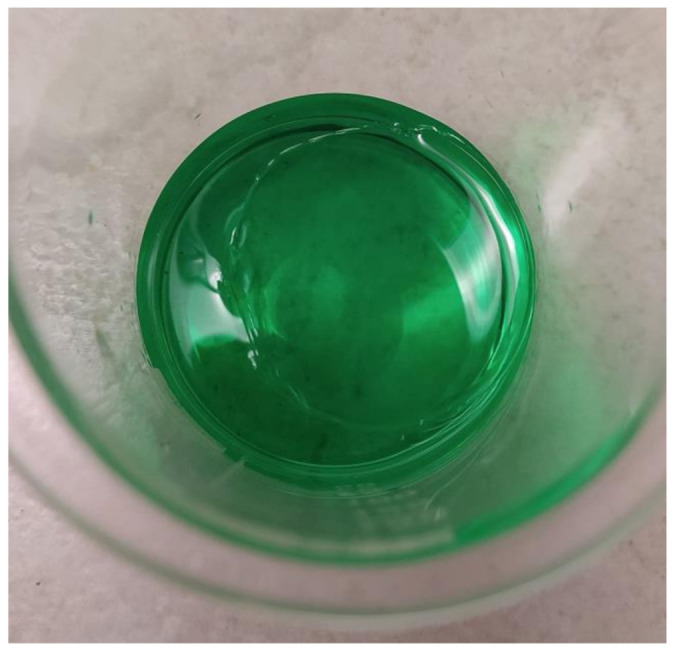
Polymer film immersed in nitric(V) acid solution.

**Figure 6:**
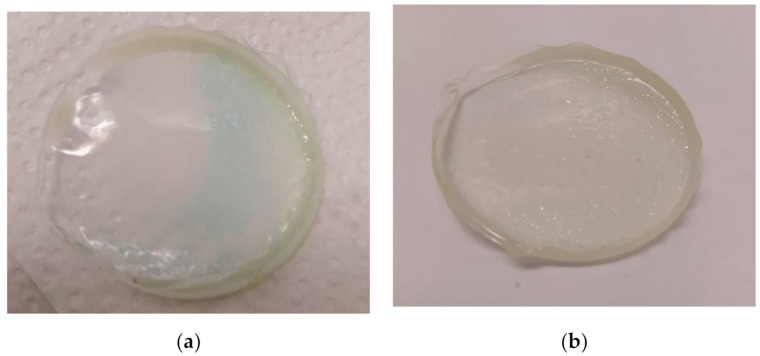
Polymer films after the first sorption process: (**a**) the membrane after removal from A solution, (**b**) the membrane after removal from B solution

The following steps were taken next:

1. A polymer film (Figure 5) was placed in 45 mL of nitric acid(V) solution (*A*) for 24 h to bind the metal ions present in the solution on its surface. Samples of the solution were taken at set intervals.
2. At the same time, a polymer film was put into 38 mL of aqua regia solution (*B*) for 24 h to bind the metal ions present in the solution on its surface. Samples of the solution were taken at set intervals.
3. After removal from the above solutions (Figure 6a,b), the polymer films were air-dried and placed in 10 mL of 5 M nitric acid for 24 h to desorb the metal ions bound on the film surface. This step was intended to allow the films to be used in subsequent sorption and desorption cycles.

To carry out further cycles of metal sorption/desorption onto/from the polymer films, the computer pins were again dissolved, and solutions *A* and *B* were prepared (in line with the “Leaching e-waste” subsection). For each new solution, the number of metal ions present was determined. Polymer films, which were purified in 5 M nitric (V) acid, were placed in the solutions thus prepared to bind the metal ions present in the solution on their surface. Again, the solution was sampled at set intervals. A total of three consecutive cycles of sorption and desorption were carried out. The repeated cycles on the polymer films that were used several times were intended to establish the possibility of their suitability for repeated application.

The concentrations of metal ions in all probes from the sorption and desorption processes were determined with inductively coupled plasma mass spectrometry (ICP-MS, NexION 300d).

## 3. Results and Discussion

**Figure 7:**
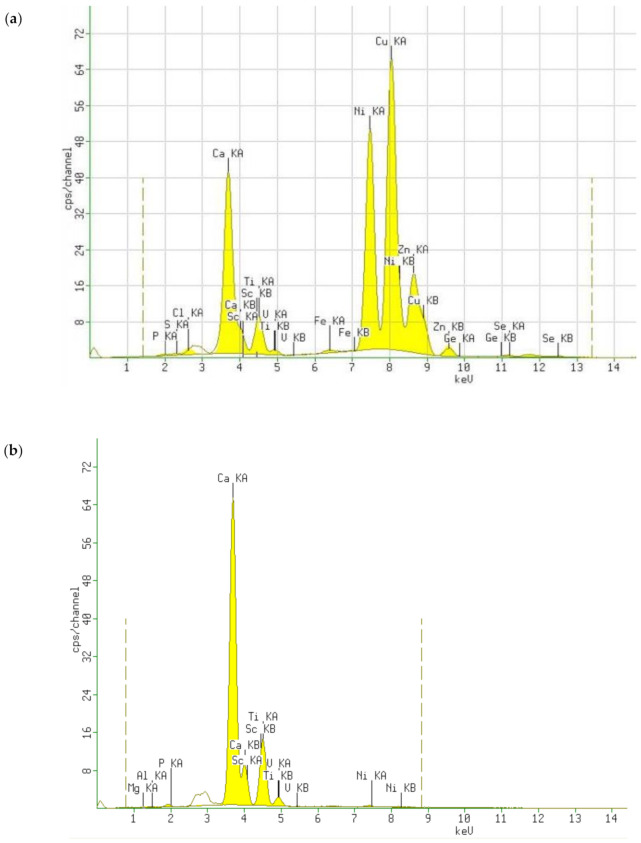
XRF spectra of (**a**) harvested computer pins; (**b**) blank test (X-ray film sample cell).

The conductive material used in electronics is usually copper. In addition, conductive elements of nickel, silver, gold, tin, or a zinc–lead alloy are applied to the surfaces of the boards. According to the above data, it was first decided to obtain the XRF qualitative spectrum of the tested computer pins. The obtained spectrum (Figure 7a) confirmed the presence of some of these elements on the surface of the tested material, which was obtained from electro-scrap. Figure 7b is, in turn, a comparative test, i.e., it is the spectrum of an empty vessel with a special X-ray film. After approximately estimating the area of the peaks obtained in spectrum 7a, which correspond to specific elements, it can be concluded that the samples contained large amounts of copper, nickel, and zinc.

To consider the above results and high copper(II) content, the tested pins had to be dissolved in nitric(V) acid first. Copper is a semi-precious metal, which means that it does not react with non-oxidizing acids (no signs of reaction with HCl). However, Cu reacts with oxidizing acids (e.g., HNO_3_).

Nitric acid and its salts are powerful oxidants—therefore, it reacts violently with metals that do not displace hydrogen (e.g., copper or silver). It also oxidizes certain non-metals, such as sulfur, carbon, and phosphorus. In addition, metals such as aluminum, chromium, and iron are passivated on contact with concentrated nitric(V) acid.

The course of the reaction of the selected divalent metals found in computer pins with concentrated nitric acid is as follows:

M^0^ + 4 HNO_3conc._ →M(NO_3_)_2_ + 2 NO_2_ + 2 H_2_O (1)

where M = Co, Ni, Cu, Zn, Sn, Pb.

In the case of monovalent silver, the course of the reaction is described by Equation (2).

Ag^0^ + 2 HNO_3conc._ → AgNO_3_ + NO_2_ + H_2_O (2)

In the system studied here, it was observed that the nitric(V) acid dissolved the above metals, as well as the silver, leaving the remaining undissolved metals at the bottom of the vessel in the form of lumps or a brownish sludge. Gold that was not dissolved in HNO_3_ accumulated on the surface of the solution in the form of flakes. The gold flakes were separated with simple centrifugation methods.

The rest of the precipitate was dissolved in another solvent. For this purpose, a mixture of nitric(V) acid and hydrochloric acid (in a 1:3 ratio), which formed the so-called aqua regia, was used. Its powerful oxidizing properties were evidenced by the fact that it dissolved all precious metals. In addition to gold, metals such as platinum, palladium, hafnium, molybdenum, and zirconium also reacted. When hot, chromium, tungsten, tantalum, rhodium, and osmium also undergo this reaction. The freshly prepared mixture was colorless but began to take on an orange–amber color after only a few minutes. This happened through the formation of nitrosyl chloride according to the equation below:

3 Cl^−^ + NO_3_ + 4 H_3_O^+^ → NOCl + 2 Cl^0^ + 6 H_2_O (3)

The reaction of gold and palladium with aqua regia occurred in two steps, as shown in Equations (4) and (5), then (6) and (7):

Au^0^ + 3 NO_3_^−^ + 6 H_3_O^+^ → Au^3+^ + 3 NO_2_ + 9 H_2_O (4)

Au^3+^ + 4Cl^−^ → [AuCl_4_]^−^ (5)

Pd^0^ + 2 NO_3_^−^ + 4 H_3_O^+^ → Pd^2+^ + 2 NO_2_ + 6 H_2_O (6)

Pd^2+^ + 4 Cl^−^ → [PdCl_4_]^2−^ (7)

### 3.1. Results of Leaching of E-Waste

After the dissolution of the computer pins in nitric(V) acid (*A*) and aqua regia (*B*), the resulting solutions were analyzed for their contents of the selected metals with the ICP-MS method. The experiments were repeated and standard deviations were calculated. Table 1 shows the results of the determination of the metal content in the freshly prepared solutions.

From the data obtained, non-ferrous metals are the largest component of computer pins. Understandably, the studied material contains significantly fewer precious metals. The metal content can be ranked as follows:

Cu(II) > Ni(II) > Zn(II) > Sn(II) > Pb(II) > Au(III) > Co(II) > Pd(II) > Ag(I) > Ta(V).

### 3.2. Results of Recovery of Metal Ions on Polymer Films Containing Cyphos IL 101

Because previously published research results have shown that Cyphos IL 101 can be successfully used to recover gold from low metal concentrations [28] and that it works well in an acidic environment [34], we decided to use this particular ionic liquid to recover metal ions from the acidic solution obtained by leaching the computer pins with nitric acid and aqua regia. Additionally, ionic liquids also perfectly fit into the assumptions of green chemistry because they are a “green” alternative to the popular volatile, flammable, and often toxic organic solvents [35]. Their use eliminates the emission of harmful substances into the natural environment because they are characterized by a practically immeasurable vapor pressure under moderate conditions [36,37].

The mechanism of the sorption process is related to the binding of the metal ions present in the solution to the Cyphos IL 101 molecule as a result of a complexation reaction. In the first stage, in an acidic solution, the proton detaches from the Cyphos IL 101 molecule, which allows the metal ion to attach to this site and form a complex compound. The prepared polymer films were immersed in solutions A and B. From this point onwards, samples were taken from each solution at specified intervals to assess the progress of the sorption process, i.e., to determine the content of metals not yet adsorbed on the polymer surface at any given time. The experiments of the sorption processes were repeated and standard deviations were calculated (Table 2 and Table 3).

The data in the above tables show that the best recovery occurred for the precious metals contained in the examined e-waste. In contrast, the recovery of non-ferrous metals (Co, Ni, Cu, Zn, Sn, Pb) occurred at 25–40%, as between 59 and 76% of these metals remained in the nitric(V) acid solution (*A*) and the aqua regia solution (*B*). This was most likely due to the selectivity of the metal ion binding agent found in the polymer films used, namely Cyphos IL 101.

This argument appears to be correct because when comparing the recovery of cobalt and gold, it is clear that Cyphos IL 101 performed more efficiently with the precious metal. Cobalt recovered from nitric(V) acid and aqua regia amounted only to 23 and 40%, respectively, while as much as 98.9% of the gold was recovered. The sorption process described could also be influenced by the composition of the solution that was studied. There are numerous examples in the literature of the use of Cyphos IL 101 for the recovery of various precious metal ions, e.g., palladium [38], gold [28], or platinum [29]. The authors of this publication point out that the effectiveness of this compound is closely related to the composition of the medium from which the metal recovery is carried out.

**Figure 8:**
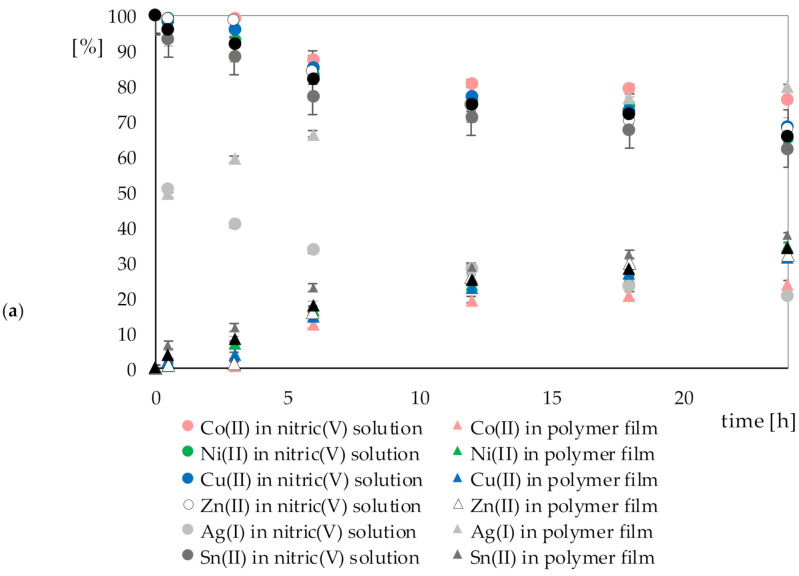

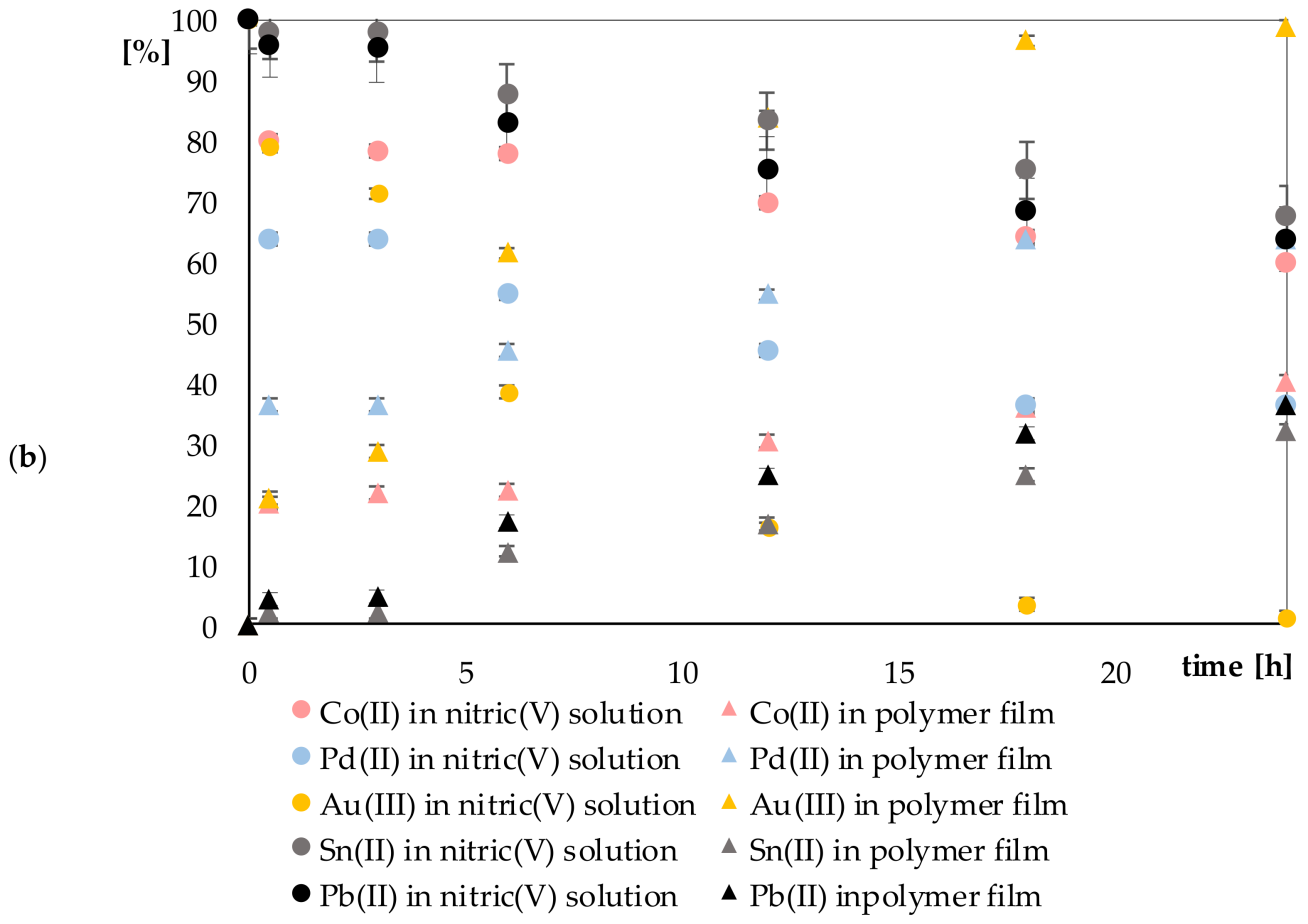
Percentage change in metal ion concentrations during the sorption process in (**a**) nitric(V) acid solution *A* and a polymer film containing Cyphos IL 101 immersed in it, as well as (**b**) aqua regia *B* and a polymer film containing Cyphos IL 101 immersed in it.

Figure 8a,b show the percentage change in metal ion concentrations during the sorption process in nitric(V) acid (*A*) and aqua regia (*B*), respectively, with the polymer film.

Only in the case of Au(III) ions can it be seen that it was possible to remove them almost completely from the solution. The amount of gold bound by the polymer films was proportional to the amount of gold lost in the solution. In the case of other metal ions, partial sorption was seen on the polymer films.

To describe the efficiency of removing metal ions from the aqueous solution using polymer films containing Cyphos IL 101, and based on the obtained results, the accumulation factor (AF) was calculated (Equation (8)). The mentioned parameter is described as follows:

(8) $$AF=\frac{c_{membrane}}{c_{0}}\cdot 100\%$$

where: *c*_0_—the initial concentration of metal ions in the feed phase (mol/dm^3^); *c_membrane_*—the concentration of metal ions in the membrane phase (mol/dm^3^).

**Figure 9:**
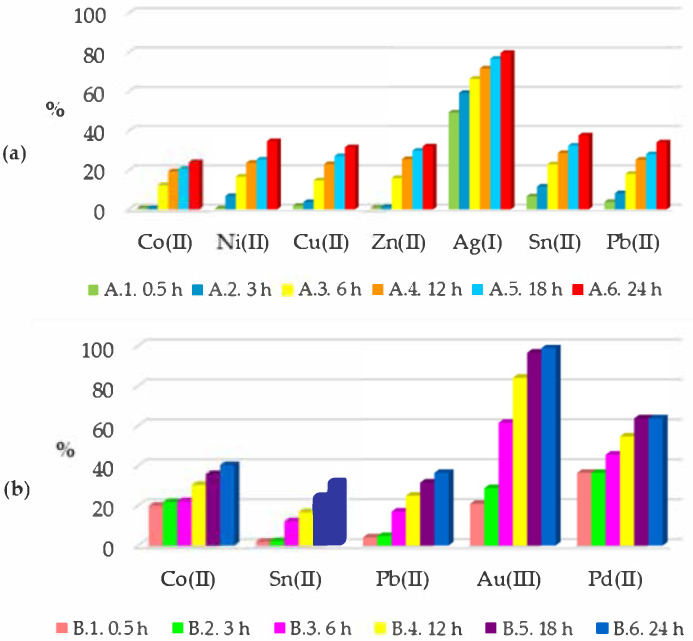
The accumulation factors describing the process of metal ions’ sorption in (**a**) nitric(V) acid (*A*) and (**b**) aqua regia (*B*) on polymer films.

From the data shown in the graphs above (Figure 9), it can be seen that the sorption process started immediately when the polymer films were dropped into the solutions. The sorption process could be quantified after only 0.5 h. For most metal ions, the accumulation factor after this time was still low, but in the case of sorption of silver, palladium, and gold ions, it was already 49.30%, 36.36%, and 20.84%, respectively. With time, all accumulation factors increased, and after 24 h, the non-precious metals did not exceed 40%. During the 24 h cycles of sorption, no decreases in accumulation factors were observed. This proved that Cyphos IL 101, which was used in the polymer films, bound the metal ions from the solution very strongly.

The sorption capacity of polymer films with 20 wt.% of Cyphos IL 101 was calculated using Equation (9):

(9) $$q_{t}=\left(\;\frac{c_{0}-c}{m}\right)\cdot V$$

where: *q_t_*—the sorption capacity (mg/g), *V*—the solution volume (dm^3^), *c*—the concentration of metal ions in the feed phase after time *t* (mol/dm^3^), and *m* —the mass of the sorbent (g).

The results of the sorption capacity parameter after 24 h of sorption are presented in Table 4.

The sorption capacity is a very important parameter of all sorbents. The sorption capacity of the studied polymer films for metal ions was higher in nitric(V) acid than in aqua regia. The highest and the lowest sorption capacities was obtained for Cu(II) and Pd(II); they were 4.91 and 3.05 × 10^−5^ mg/g, respectively. The low values of sorption capacities for precious metal ions were caused by their small amounts in the studied WEEE. The received results can be compared with those found in the literature; e.g., the sorption capacities of materials that were obtained through impregnation of Cyphos IL101 on Florisil and Silica were 2.94 and 3.97 mg/g, respectively [39]. The sorption capacity of Amberlite XAD-7 with immobilized Cyphos IL 101 was, in turn, 71 mg/g [40], but the costs of preparing these resins were higher than those of the polymer films studied in this paper.

As a result of the sorption process on polymer films containing 20 wt.% of Cyphos IL 101, the following amounts of metals were recovered from 0.5 g of the computer pins studied (Table 5).

Based on the obtained results, it was found that using a polymer film containing 20 wt.% of Cyphos IL 101 as an ion carrier can recover copper(II) (4.28454 mg) > nickel(II) (3.73884 mg) > gold(III) (0.36179 mg) > tin(II) (0.23698 mg) and trace amounts of Pb(II) > Co(II) > Ag(I) > Pd(II) from 0.5 g e-waste. This allowed the estimation of the number of metals in a tonne of e-waste (Table 6).

The quantity of metals recovered with the polymer films appears to be small. However, an average Pole produces around 12 kg of e-waste per year, which amounts to almost 500,000 tonnes nationwide, which is around 77% of all waste of this type generated in the European Union. This number is so high because new sub-groups of e-waste that already exist in Western Europe are now beginning to emerge in Poland. The consequence of this phenomenon will be a continuous and dynamic increase in the amount of e-waste produced in our country. In contrast, the US produces the most electronic waste worldwide, according to a United Nations report. Western countries are also unable to recycle 100% of their e-waste, which is why they primarily transport it to African and Asian countries, as the cost of transport to these countries is twice as low as recycling on site [41].

The recovery method proposed in this paper is a good alternative for recycling valuable metals from electronic waste. Furthermore, it should be noted that this method allows for very selective recovery of precious metals because the percentage that was successfully recovered in relation to other non-ferrous metals was high (98.9% Au(III), 79.3% Ag(I), and 63.6% Pd(II)).

### 3.3. Evaluation of the Long-Term Suitability of Polymer Films Containing Cyphos IL101

Polymer films containing Cyphos IL 101 were regenerated after each sorption process by immersion in 5 M HNO_3_ solution. The acid solution used for this process was also analyzed for the presence of the metals studied. The polymer films thus cleaned were placed again in solutions *A* and *B*, which were obtained by dissolving the computer pins in nitric(V) acid and aqua regia, respectively. The sorption and desorption processes were carried out on the same polymer films three times in 24 h cycles.

**Figure 10:**
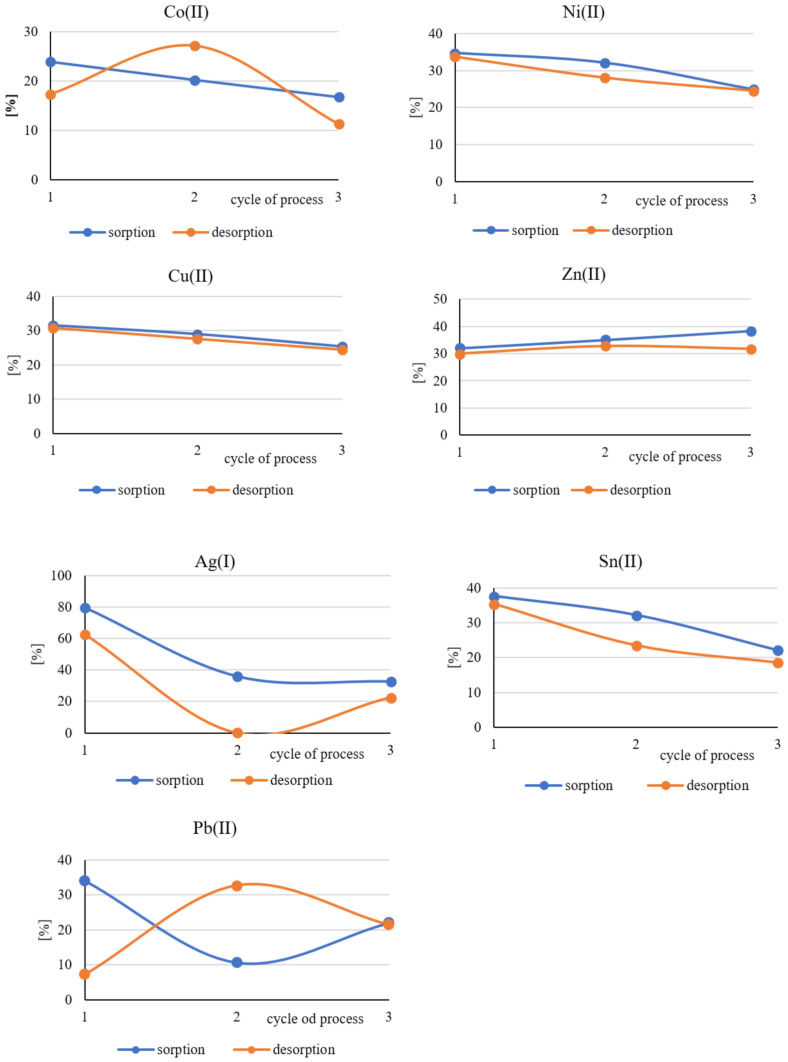
Percentage distribution of the quantities of metals adsorbed during the consecutive sorption processes on polymer films from the pin solution dissolved in concentrated nitric(V) acid *A* and desorbed from their surfaces during consecutive processes of desorption.

**Figure 11:**
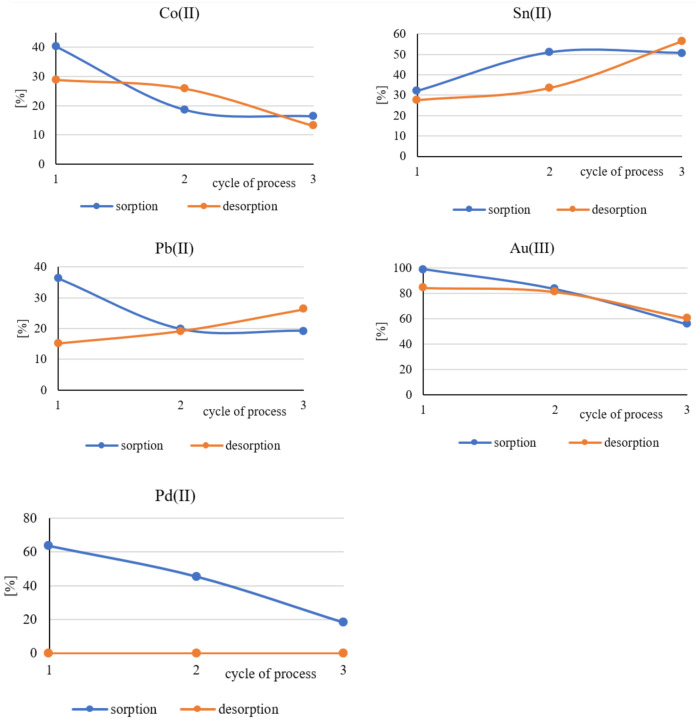
Percentage distribution of the quantities of metals adsorbed during the consecutive sorption processes on polymer films from the pin solution dissolved in aqua regia *B* and desorbed from their surfaces during consecutive processes of desorption.

Figure 10 and Figure 11 show the percentage distribution of the quantities of metals adsorbed on polymer films during the consecutive sorption processes or desorbed from their surface during desorption.

The above graphs show that the polymer films used in this study can still bind metal ions from the solutions formed by dissolving the computer pins even after three consecutive sorption and desorption cycles. The obtained results are not unambiguous. It can be only seen that in most cases, the metal uptake by polymer films decreases with the next cycle of sorption. This may be related to the phenomenon of film clogging, which is caused by incomplete regeneration of the surface during consecutive desorption processes, during which decreasing quantities of metal ions are released into the solution. In the case of palladium, the number of desorbed ions was impossible to determine. Palladium, however, was a trace element in the material studied.

## 4. Summary

Although the use of polymer films does not allow the complete recovery of all of the metals present in computer pins, it indeed allows the recovery of the most precious metals. An undoubtable advantage of the materials used in this study is that they can be reused several times. The obtained films have more benefits than the other more traditional methods (e.g., electrowinning, solvent extraction, ionic exchange resin). First, the cost of obtaining polymer films is much lower than the cost of ion exchange resins because synthesis is provided by using simple and cheap substrates (commonly known polyvinyl chloride and plasticizer bis(2-ethylhexyl)adipate, and the easily accessible commercial metal ion carrier Cyphos IL 101), and special equipment is not required. Moreover, no power source is required to carry out the process of metal ion recovery. For example, the electrowinning methods need lots of power. All compounds added to polymer films are eco-friendly, so the material is safe for the natural environment and it does not cause pollution compared with solvent extraction, in which toxic solvents need to be used. Finally, the most important benefit of polymer films is that they can be easily separated from the solution after process of sorption of metal ions.

It is also important that in the described procedure of preparing e-waste samples for metals ion recovery, there is no need to use complex pre-treatments. For example, one alternative non-contaminating reagent used for gold leaching is thiourea [42], but as reported by Ippolito et al. [43], recovering gold from printed circuit boards or spent cell phones “as is” for thiourea leaching was not feasible due to the low Au grade release and recovery of only 15%. Direct leaching of thiourea onto the plate failed due to the competition between copper and gold for thiourea to form the corresponding complexes. Only the use of various pretreatment methods made it possible to dissolve the copper and increase the recovery of gold to about 80%.

The method proposed in this paper meets the requirements of a process that is carried out in the cheapest possible way and, at the same time, allows high efficiency with low energy and material inputs.

## 5. Conclusions

The results concerning the application of polymer films containing Cyphos IL 101 for the recovery of various metal ions ((Ni(II), Zn(II), Co(II), Cu(II), Sn(II), Pb(II), Ag(I), Pd(II), and Au(III)) from computer e-waste clearly show that the ionic liquid used allows for the effective recovery of noble metal ions. The efficiency of the recovery process was as follows: 98.9% for gold, 79.3% for silver, and 63.6% for palladium. In the case of heavy metal ion recovery, the efficiency was much lower, ranging from 25 to 40%. Moreover, the results of the experiments also demonstrate that the polymer films used in this research can still bind metal ions from solutions created by leaching computer pins even after three consecutive sorption and desorption cycles. In most cases, the metal uptake by polymer films decreases after each cycle. Due to the very high efficiency of gold ion recovery when using the ionic liquid Cyphos IL 101 and the high cost of this metal, the procedure described in this paper may be a good alternative to other less efficient, more complex, and costly recovery solutions.

## Figures and Tables

**Table 1:** Metal content found in freshly prepared solutions formed by dissolving computer pins: in nitric(V) acid (*A*) and aqua regia (*B*).

Symbol of the Sample	Co(II)	Ni(II)	Cu(II)	Zn(II)	Ag(I)	Sn(II)	Pb(II)	Au(III)	Pd(II)	Ta(V)	
		(ppb)									
A.0	35.1	239,656.29	301,789.35	102,075.88	4.32	3065.40	3262.64	-	-	BLQ	
B.0	22.1	-	-	-	-	15,111.88	2309.07	47.63	0.11	BLQ	

BLQ—beyond the limit of quantification. The given values of the concentration carry ± 0.01 ppb.

**Table 2:** Changes in the values of metal ion concentrations in nitric(V) acid (*A*) in solution with sorption time on polymer film.

Symbol of the Sample	Time (h)	Co(II)	Ni(II)	Cu(II)	Zn(II)	Ag(I)	Sn(II)	Pb(II)
		(ppb)						
A.1	0.50	34.92	238,563.29	296,386.02	101,145.38	2.19	2863.98	3139.67
A.2	1.50	34.94	223,442.34	290,767.62	100,853.95	1.76	2712.10	2996.41
A.3	3.00	30.83	199,932.97	257,349.24	85,830.85	1.45	2363.87	2676.53
A.4	10.00	28.31	183,021.32	232,285.68	75,987.33	1.22	2185.95	2438.11
A.5	23.00	27.91	178,906.54	220,067.10	71,645.99	1.01	2072.34	2347.74
A.6	24.00	26.72	156,570.95	206,577.27	69,413.90	0.88	1911.74	2152.41
		**76.07%**	**65.33%**	**68.45%**	**68.00%**	**20.37%**	**62.36%**	**65.97%**

The given values of the concentrations carry ± 0.01 ppb.

**Table 3:** Changes in the values of metal ion concentrations in aqua regia (*B*) in solution with sorption time on polymer film.

Symbol of the Sample	Time (h)	Co(II)	Sn(II)	Pb(II)	Au(III)	Pd(II)
		(ppb)				
B.1	0.50	1.77	14,823.34	2215.05	37.70	0.07
B.2	1.50	1.73	14,783.23	2200.64	33.95	0.07
B.3	3.00	1.72	13,281.13	1917.68	18.37	0.06
B.4	10.00	1.54	12,586.45	1734.79	7.64	0.05
B.5	23.00	1.42	11,367.46	1582.00	1.65	0.04
B.6	24.00	1.32	10,241.67	1469.91	0.54	0.04
		**59.73%**	**67.77%**	**63.66%**	**1.13%**	**36.36%**

The given values of the concentration carry ± 0.01 ppb.

**Table 4:** The sorption capacity of the materials used.

Metal Ions	q_t_ (mg/g) in *A* Solution	q_t_ (mmol/g) in *A* Solution	q_t_ (mg/g) in *B* Solution	q_t_ (mmol/g) in *B* Solution
Co(II)	4.33 × 10^−4^	7.35 × 10^−7^	3.87 × 10^−5^	6.57 × 10^−7^
Ni(II)	4.28	7.30 × 10^−2^	-	-
Cu(II)	4.91	7.72 × 10^−2^	-	-
Zn(II)	1.68	2.57 × 10^−2^	-	-
Ag(I)	1.77 × 10^−4^	1.64 × 10^−6^	-	-
Sn(II)	5.95 × 10^−2^	5.01 × 10^−4^	2.12 × 10^−2^	1.78 × 10^−3^
Pb(II)	5.72 × 10^−2^	2.76 × 10^−4^	3.65 × 10^−2^	1.76 × 10^−4^
Au(III)	-	-	2.05 × 10^−3^	1.04 × 10^−5^
Pd(II)	-	-	3.05 × 10^−^^5^	2.86 × 10^−8^

The given values of the qt carry ± 0.01 mg/g.

**Table 5:** The total amounts of metal ions recovered from computer e-waste using polymer films.

	Co(II)	Ni(II)	Cu(II)	Zn(II)	Ag(I)	Sn(II)	Pb(II)	Au(III)	Pd(II)
mg of Metal Ions/0.5 g Electro-Scrap	0.00038	3.73884	4.28454	1.46979	0.00015	0.23698	0.08185	0.00179	0.000002

The given values of the total amounts of recovered metal ions ± 0.00001 mg/g.

**Table 6:** Estimated amounts of metals recovered using the method proposed in this paper per tonne of e-waste.

	Co(II)	Ni(II)	Cu(II)	Zn(II)	Ag(I)	Sn(II)	Pb(II)	Au(III)	Pd(II)
mg of Metal Ions/0.5 g Electro-Scrap	0.00076	7.47768	8.56909	2.93958	0.00031	0.47397	0.16369	0.72358 *	0.000005

* This value also includes the amount of gold obtained during the leaching of e-waste in nitric(V) acid. The given values of the total amounts of recovered metal ions ± 0.00001 mg/g.

## Data Availability

Not applicable.

## References

[1] Zeng X., Gong R., Chen W.-Q., Li J. (2016). Uncovering the Recycling Potential of “New” WEEE in China. Environ. Sci. Technol..

[2] Hamdan S., Saidan M.N. (2020). Estimation of E-waste Generation, Residential Behavior, and Disposal Practices from Major Governorates in Jordan. Environ. Manag..

[3] Shaikh S., Thomas K., Zuhair S. (2020). An exploratory study of e-waste creation and disposal: Upstream considerations. Resour. Conserv. Recycl..

[4] Directive 2002/96/EC of the European Parliament and of the Council of 27 January 2003 on Waste Electrical and Electronic Equipment (WEEE). https://eur-lex.europa.eu/legal-content/EN/TXT/?uri=celex%3A32002L0096.

[5] Sethurajan M., van Hullebusch E.D., Fontana D., Akcil A., Deveci H., Batinic B., Leal J.P., Gasche T.A., Kucuker M.A., Kuchta K. (2019). Recent advances on hydrometallurgical recovery of critical and precious elements from end of life electronic wastes—A review. Crit. Rev. Environ. Sci. Technol..

[6] Akcil A., Agcasulu I., Swain B. (2019). Valorization of waste LCD and recovery of critical raw material for circular economy: A review. Resour. Conserv. Recycl..

[7] Anand A., Jha K.J., Kumar V., Sahu R. Recycling of precious metal gold from waste electrical and electronic equipments (WEEE): A review. Proceedings of the XIII International Seminar on Mineral Processing Technology.

[8] Li W., Achal V. (2020). Environmental and health impacts due to e-waste disposal in China—A review. Sci. Total. Environ..

[9] Sun Z., Cao H., Xiao Y., Sietsma J., Jin W., Agterhuis H., Yang Y. (2017). Toward Sustainability for Recovery of Critical Metals from Electronic Waste: The Hydrochemistry Processes. ACS Sustain. Chem. Eng..

[10] Hagelüken C., Corti C.W. (2010). Recycling of gold from electronics: Cost-effective use through ‘Design for Recycling’. Gold Bull..

[11] Pasiecznik I., Banaszkiewicz K., Syska Ł. (2017). Local community e-waste awareness and behavior. Polish case study. Environ. Prot. Eng..

[12] Kornacki W. (2002). Recykling płytek drukowanych. II Krajowa Konferencja Naukowo-Techniczna “Ekologia w Elektronice”.

[13] Ibanescu D., Gavrilescu D.C., Teodosiu C., Fiore S. (2018). Assessment of the waste electrical and electronic equipment management systems profile and sustainability in developed and developing European Union countries. Waste Manag..

[14] Fizaine F. (2020). The economics of recycling rate: New insights from waste electrical and electronic equipment. Resour. Policy.

[15] Bahubalendruni M.V.A.R., Varupala V.P. (2020). Disassembly Sequence Planning for Safe Disposal of End-of-Life Waste Electric and Electronic Equipment. Natl. Acad. Sci. Lett..

[16] Tanskanen P. (2013). Management and recycling of electronic waste. Acta Mater..

[17] Doidge E.D., Carson I., Tasker P.A., Ellis R.J., Morrison C.A., Love J.B. (2016). A Simple Primary Amide for the Selective Recovery of Gold from Secondary Resources. Angew. Chem. Int. Ed..

[18] Kaya M. (2016). Recovery of metals and nonmetals from electronic waste by physical and chemical recycling processes. Waste Manag..

[19] Rao M.D., Singh K.K., Morrison C.A., Love J.B. (2020). Challenges and opportunities in the recovery of gold from electronic waste. RSC Adv..

[20] Assadian M., Idris M.H., Shahri S.M.G., Gholampour B. (2013). Gold Recovery from WEEE by Chlorine System. Appl. Mech. Mater..

[21] Rohit I., Dhanunjaya M., Arunabh R., Himanshu M., Verma R., Singh K.K. (2020). Potential of polymer inclusion membrane process for selective recovery of metal values from waste printed circuit boards: A review. J. Clean. Prod..

[22] Kavitha N., Palanivelu K. (2012). Recovery of copper(II) through polymer inclusion membrane with di(2-ethylhexyl) phosphoric acid as carrier from e-waste. J. Membr. Sci..

[23] Kubota F., Kono R., Yoshida W., Sharaf M., Kolev S.D., Goto M. (2019). Recovery of gold ions from discarded mobile phone leachate by solvent extraction and polymer inclusion membrane (PIM) based separation using an amic acid extractant. Sep. Purif. Technol..

[24] Miguel E.R.D.S., Garduño-García A.V., Aguilar J.C., de Gyves J. (2007). Gold(III) Transport through Polymer Inclusion Membranes: Efficiency Factors and Pertraction Mechanism Using Kelex 100 as Carrier. Ind. Eng. Chem. Res..

[25] Bonggotgetsakul Y.Y.N., Cattrall R.W., Kolev S.D. (2016). Recovery of gold from aqua regia digested electronic scrap using a poly(vinylidene fluoride-co-hexafluoropropene) (PVDF-HFP) based polymer inclusion membrane (PIM) containing Cyphos^®^ IL 104. J. Membr. Sci..

[26] Nowik-Zajac A., Zawierucha I., Kozlowski C., Nowik-Zając A. (2019). Selective removal of silver(I) using polymer inclusion membranes containing calixpyrroles. RSC Adv..

[27] Bahrami S., Yaftian M.R., Najvak P., Dolatyari L., Shayani-Jam H., Kolev S.D. (2020). PVDF-HFP based polymer inclusion membranes containing Cyphos^®^ IL 101 and Aliquat^®^ 336 for the removal of Cr(VI) from sulfate solutions. Sep. Purif. Technol..

[28] Campos K., Vincent T., Bunio P., Trochimczuk A., Guibal E. (2008). Gold Recovery from HCl Solutions using Cyphos IL-101 (a Quaternary Phosphonium Ionic Liquid) Immobilized in Biopolymer Capsules. Solvent Extr. Ion Exch..

[29] Vincent T., Parodi A., Guibal E. (2008). Pt recovery using Cyphos IL-101 immobilized in biopolymer capsules. Sep. Purif. Technol..

[30] Regel-Rosocka M., Rzelewska M., Baczynska M., Janus M., Wisniewski M. (2015). Removal of palladium(II) from aqueous chloride solutions with Cyphos phosphoniumionic liquids as metal ion carriers for liquid-liquid extraction and transport across polymer inclusion membranes. Physicochem. Probl. Miner. Process..

[31] Pospiech B. (2015). Application of Phosphonium Ionic Liquids as Ion Carriers in Polymer Inclusion Membranes (PIMs) for Separation of Cadmium(II) and Copper(II) from Aqueous Solutions. J. Solut. Chem..

[32] Nowik-Zajac A., Zawierucha I., Kozlowski C. (2020). Selective Transport of Ag(I) through a Polymer Inclusion Membrane Containing a Calix[4]pyrrole Derivative from Nitrate Aqueous Solutions. Int. J. Mol. Sci..

[33] Radzyminska-Lenarcik E., Ulewicz M., Pyszka I. (2020). Application of Polymer Inclusion Membranes Doped with Alkylimidazole to Separation of Silver and Zinc Ions from Model Solutions and after Battery Leaching. Materials.

[34] Yaftian M.R., Almeida M.I.G., Cattrall R.W., Kolev S.D. (2018). Selective extraction of vanadium(V) from sulfate solutions into a polymer inclusion membrane composed of poly(vinylidenefluoride-co-hexafluoropropylene) and Cyphos^®^ IL 101. J. Membr. Sci..

[35] Burczyk B. (2014). Green Chemistry Outline.

[36] Earle M.J., Esperança J.M., Gilea M.A., Lopes J.N.C., Rebelo L.P., Magee J.W., Seddon K.R., Widegren J.A. (2006). The distillation and volatility of ionic liquids. Nat. Cell Biol..

[37] Ludwig R. (2001). Water: From Clusters to the Bulk. Angew. Chem. Int. Ed..

[38] Cieszyńska A., Regel-Rosocka M., Wiśniewski M. (2007). Extraction of Palladium(II) Ions from Chloride Solutions with Phosphonium Ionic Liquid Cyphos^®^ IL101. Pol. J. Chem. Technol..

[39] Negrea A., Lupa L., Ciopec M., Negrea P. (2013). Characterization of Strontium Adsorption from Aqueous Solutions Using Inorganic Materials Impregnated with Ionic Liquid. Int. J. Chem. Eng. Appl..

[40] Navarro R., Saucedo I., Gonzalez C., Guibal E. (2012). Amberlite XAD-7 impregnated with Cyphos IL-101 (tetraalkylphosphonium ionic liquid) for Pd(II) recovery from HCl solutions. Chem. Eng. J..

[41] Maj R. (2015). Analiza Niemieckiego System Recyklingowego Elektroodpadów Oraz Uwarunkowania Prawno-Gospodarcze Wprowadzenia Go w Polsce.

[42] Juarez C., Dutra A. (2000). Gold electrowinning from thiourea solutions. Miner. Eng..

[43] Ippolito N.M., Medici F., Pietrelli L., Piga L. (2021). Effect of Acid Leaching Pre-Treatment on Gold Extraction from Printed Circuit Boards of Spent Mobile Phones. Materials.

